# Ni Nanoparticles Embedded Ti_3_C_2_T*_x_*-MXene Nanoarchitectures for Electrochemical Sensing of Methylmalonic Acid

**DOI:** 10.3390/bios12040231

**Published:** 2022-04-10

**Authors:** Jai Kumar, Razium Ali Soomro, Rana R. Neiber, Nazeer Ahmed, Shymaa S. Medany, Munirah D. Albaqami, Ayman Nafady

**Affiliations:** 1 State Key Laboratory of Organic-Inorganic Composites Beijing Key Laboratory of Electrochemical Process and Technology for Materials, Beijing University of Chemical Technology, Beijing 100029, China; 2020420024@mail.buct.edu.cn; 2 Beijing Key Laboratory of Ionic Liquids Clean Process, CAS Key Laboratory of Green Process and Engineering, Institute of Process Engineering, Chinese Academy of Sciences, Beijing 100190, China; rana@ipe.ac.cn; 3 College of Chemical Engineering, University of Chinese Academy of Sciences, 19A Yuquan Road, Beijing 100049, China; 4 Research Center on Nanotechnology Applied to Engineering of Sapienza (CNIS), Sapienza University of Rome, P. le Aldo Moro 5, 00185 Rome, Italy; ahmed.1875130@studenti.uniroma1.it; 5 Department of Chemistry, Faculty of Science, Cairo University, Giza 12613, Egypt; shymaaamir80@cu.edu.eg; 6 Department of Chemistry, College of Science, King Saud University, P.O. Box 2455, Riyadh 11451, Saudi Arabia; muneerad@ksu.edu.sa (M.D.A.); anafady@ksu.edu.sa (A.N.)

**Keywords:** MXenes, methylmalonic acid, Ni NPs, electrochemical sensors, vitamin B12

## Abstract

MXenes-Ti_3_C_2_T*_x_*, based on their versatile surface characteristics, has rapidly advanced as an interactive substrate to develop electrochemical sensors for clinical applications. Herein, Ni embedded Ti_3_C_2_T*_x_* (MX−Ni) composites were prepared using a self-assembly approach where Ti_3_C_2_T*_x_* sheets served as an interactive conductive substrate as well as a protective layer to nickel nanoparticles (Ni NPs), preventing their surface oxidation and aggregation. The composite displayed a cluster-like morphology with an intimate interfacial arrangement between Ni, Ti_3_C_2_T*_x_* and Ti_3_C_2_T*_x_*-derived TiO_2_. The configuration of MX−Ni into an electrochemical sensor realized a robust cathodic reduction current against methylmalonic acid (MMA), a biomarker to vitamin B12 deficiency. The synergism of Ni NPs strong redox characteristics with conductive Ti_3_C_2_T*_x_* enabled sensitive signal output in wide detection ranges of 0.001 to 0.003 µM and 0.0035 to 0.017 µM and a detection sensitivity down to 0.12 pM of MMA. Importantly, the sensor demonstrated high signal reproducibility and excellent operational capabilities for MMA in a complex biological matrix such as human urine samples.

## 1. Introduction

Methylmalonic acid (MMA) is an important biomarker of vitamin B12 due to its role in converting L-methyl malonyl-CoA into succinyl-CoA. In case of B12 deficiency, the body’s ability to convert L-methyl malonyl-CoA to succinyl-CoA is hindered, resulting in the rise of MMA concentration in blood serum and urine [1,2,3]. MMA level could rise in the early stage of vitamin B12 deficiency, despite B12 being normal (190–200 pg·mL^−1^) [4]. Thus, quantifying MMA could be used as a functional indicator to even a slight deficiency of B12, which a standard B12-test might not assess. The normal physiologically levels of MMA are presently set below 370 nmol·L^−1^ or 0.37 µmol·L^−1^ and 0.4–2.5 μmol/mmol of creatinine, respectively [4]. Although the increase in MMA may suggest a B12 deficiency, the cut-off value for its disease-relevant range is still not widely agreed upon and may vary between 240 and 300 nM [5]. Thus, the precise identification and detection of MMA in the human body are of tremendous therapeutic relevance since it might imply a vitamin B12 deficiency. The direct detection and quantification of B12 in the body are challenging based on its inactivity and complex chemistries with bound proteins. Thus, to ensure certainty in quantifying vitamin B12, the MMA test is often used in conjunction with vitamin B12 [6]. MAA is also an important biomarker to the rare genetic condition known as methylmalonic academia, which occurs in infants and could lead to severe toxicity and other health issues [7,8]. Thus, a portable, low-cost and speedy method of detecting MAA might lead to an improved clinical practice with the promise of lab-free or on-spot testing.

Among the usual methods for detecting MAA, spectrophotometry and chromatography are trustworthy [9,10]. These approaches, however, are frequently time-consuming, have limited sensitivity and need well-maintained equipment and infrastructure. Electrochemical sensors using nanomaterial-based electrode systems, on the other hand, might provide better sensitivity and lower detection limits in shorter durations with minimal sample size and infrastructure needs [11].

The electrochemical sensors for clinical analysis require specific electrode materials that can offer higher current sensitivity even at low concentrations and are affordable for biological detection [12]. At present, the common materials include metal oxides, metals, carbonaceous materials such as graphene and their composites [13,14]. Since the biomarkers are often in trace levels, a robust and high signal sensitivity is essential for precise quantification. Thus, composite systems with more than one integral component have proven reliable. In the case of electrochemical sensors, graphene or its compositional analogues are prominent in attaining a dependable sensitivity with favorable immobilization and functionalization surface chemistries [15]. However, graphene and its composites often challenge electrode modification because of their lower aqueous dispersibility and complex synthesis procedures. Moreover, graphene’s toxicity and poor water dispersibility make it challenging to design composite-based modified electrodes for clinical investigation.

The recently introduced 2D MXene materials, specifically Ti_3_C_2_T*_x_,* have gained significant attention from the sensors community based on their versatile characteristics such as high aqueous dispersibility, excellent conductivity and superior surface-chemical tunability [16]. MXenes are 2D transition metal carbides, nitrides, or carbo-nitrides with a typical formula of M*_n_*_+1_X*_n_*T*_x_*. Here, “M” is a transition metal, “X” can be (C or N), *n* = 1 to 3, and “T*_x_*” can be any surface functionality, i.e., (-OH, -F, -O). Unlike graphene, MXenes may be easily obtained by selectively etching the metal layers from the MAX phase which can be an element from groups 13–14 of the periodic table, using hydrofluoric acid or a combination of lithium fluoride and hydrochloric acid [17]. MXenes’ customizable surface chemistry, non-toxicity, flexibility and quick electron transfer kinetics have resulted in the rapid growth of the MXene-composite community with a focus on their integration with metal and metal oxide nanostructures to realize surface-redox superiority that could be utilized in developing electrochemical sensors for clinical purposes [18,19,20]. 

Among metal electrocatalysts, zero-valent Ni metal nanoparticles are well-known for their robust electrochemical properties (strong redox couple), non-precious nature, low toxicity and, perhaps most significantly, ease of preparation from common precursors [21,22]. Despite their enhanced redox activity, Ni NPs often exhibit poor oxidation stability and suffer from severe aggregation, compromising their catalytic/electrocatalytic performance [23]. Here, a suitable substrate that could offer interactive support to Ni NPs without compromising their inherent chemical and surface characteristics would be viable to realize a robust composite system for clinical analysis. MXenes-Ti_3_C_2_T*_x_,* with their high aqueous dispersibility, conductivity and reducing ability, may serve as interactive support to Ni-NPs inhibiting their surface-oxidation and aggregation besides providing a conductive bed to facilitate the charge-transfer kinetics for improved electrochemical performance. 

Herein we provide a simple and efficient route to composite MXene−Ti_3_C_2_T*_x_* and Ni NPs into nanoclusters. A few-layer thick Ti_3_C_2_T*_x_* were prepared using the conventional HF-etching approach, whereas Ni NPs was prepared using a simple wet-chemical reduction method. The combination of Ni and Ti_3_C_2_T*_x_* resulted in cluster-like structures where electrostatic assembly of Ni with Ti_3_C_2_T*_x_* resulted in the crumpled-Ti_3_C_2_T*_x_* morphology. The Ni loaded Ti_3_C_2_T*_x_* (MX−Ni) was then used for the electrochemical detection of methylmalonic acid (MMA) in a deaerated aqueous PBS system. The improved surface characteristics based on the synergism of Ni and Ti_3_C_2_T*_x_* afforded high signal sensitivity in the broader detection range of 0.001 to 0.017 µM and a detection limit as low as 0.12 pM. The fabricated MX-Ni based electrochemical sensor exhibited reliable selectivity towards MAA in human urine samples, confirming its promising utilization in practical clinical applications.

The proposed use of the MXene-Ni composite system might be extended to other metals and oxides, paving a new route for enhanced electrochemical sensors with Ti_3_C_2_T*_x_* as a potential interactive conductive substrate.

## 2. Materials and Methods

### 2.1. Synthesis of Ni Nanoparticles (Ni NPs)

Zero-valent Ni NPs were synthesized using a simple wet-chemical reduction route with hydrazine hydrate as a mild reductant. In a typical experiment, a mixture of NiCl_2_ (0.05 M) and CTAB (0.012 M) was homogenized and purged with N_2_ to remove oxygen from the solution. The mixture was then gently introduced with 0.25 M of hydrazine, followed by 0.01 M of NaOH to facilitate the reduction process. The mixture was kept under seal and stirring conditions for 24 h whereby the formation of black precipitates confirmed the generation of Ni NPs. The role of CTAB was not only to cap the formed Ni metallic nuclei from aggregation but to induce cationic charge, which would later facilitate their electrostatic assembly with negatively charged Ti_3_C_2_T*_x_* surface.

### 2.2. Synthesis of Ti_3_C_2_T_x_ and MX−Ni Composite

The Ti_3_C_2_T*_x_* sheets were obtained using our previously utilized chemical etching procedure [12]. In a typical synthesis technique, the specific content of Ti_3_AlC_2_ (MAX-Phase) was etched for 24 h at a temperature of 35 °C using a combination of LiF (1.3 g) and HCl (6 M). The etching of a few-layer thick was completed by neutralizing pH and washing to remove any surface-bound impurities. The final Ti_3_C_2_T*_x_* nanosheets were re-dispersed in an N_2_-saturated aqueous system with a total content of 1.5 g·mL^−1^.

The MX−Ni composites were prepared by introducing 1.5 mL of CTAB-capped Ni NPs within the MXene mixture (1.0 g 5.0 mL^−1^). The mixture was kept under slow stirring conditions for 4 h to allow electrostatic assembly of Ni NPs over MXene sheets.

### 2.3. Characterization and Electrochemical Measurement

Transmission electron microscopy (TEM) (JEOL 2010) (JEOL, Tokyo, Japan) was used for morphological evaluation of MXene, Ni NPs and their composite structures. The compositional analysis was carried out using powder X-ray diffraction (XRD) (Cu Ka, k = 1.5406 Å; Rigaku Corporation, Tokyo, Japan with X-ray photoelectron spectroscopy (XPS) (Axis-Nova, Kratos Analytical Ltd., Manchester, UK) for surface oxidation states of compositional components of the composites. The electrochemical experiments were performed on a CHI660e electrochemical workstation equipped with a reference electrode of Ag/AgCl and counter electrodes of platinum wire. The working electrode was constructed by depositing a pre-formed MX-Ni dispersion (10 µL of MX-Ni (1.0 g 5.0 mL^−1^) in Nafion (1.5%)) over a pre-polished GCE electrode. All the experiments were recorded in a deaerated 0.1 M PBS (pH 7) electrolytic system at room temperature.

## 3. Results and Discussion

**Figure 1:**
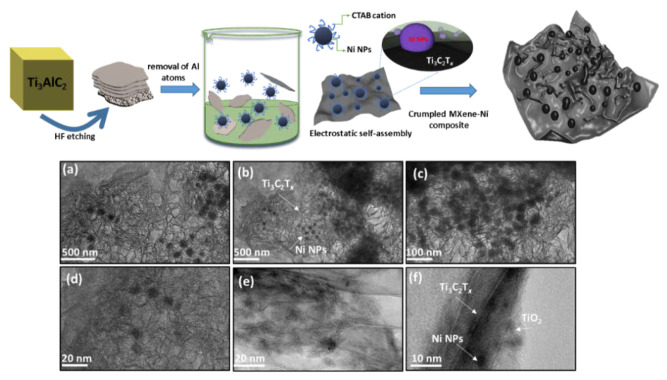
General schematic illustrating the self-assembly based formation of MX−Ni composites; (**a**–**e**) TEM images depicting Ni NPs loaded MXene cluster-like architectures with (**f**) focused TEM image showing the compact interfacial arrangement between Ti_3_C_2_T*_x_*, Ni and Ti_3_C_2_T*_x_* derived TiO_2_ particles.

The Ni NPs were encapsulated onto a few-layer thick MXene-Ti_3_C_2_T*_x_* using an electrostatic self-assembly approach. Here, cationic surfactant capped Ni NPs interacts with negatively charged surface moieties of the Ti_3_C_2_T*_x_* sheets resulting in crumpled surface structures. Figure 1 shows a general schematic of the self-assembly, where etched MXene sheets are homogenized with Ni NPs, resulting in the formation of Ni−Ti_3_C_2_T*_x_* interface (focus illustration) and cluster-like MX−Ni composite. In general, Ni NPs are prone to rapid aggregation, however, the use of MXenes as interactive substrate could inhibit the metal-metal aggregation. Moreover, the reducing nature of Ti_3_C_2_T*_x_* further resists any surface oxidation of Ni to NiO. The TEM images of pristine few-layer thick MXene−Ti_3_C_2_T*_x_* and as-prepared Ni NPs are shown in Figure S1a,b (see the Supplementary Materials), confirming Ti_3_C_2_T*_x_* Lamellar-like morphology and aggregated Ni NPs. Figure 1a–e shows the TEM images for the MX-Ni composite, where thoroughly loaded Ni NPs could be seen onto MXene sheets. The loaded Ni NPs are highly dispersed in nature without any structural damage or aggregation and possess a homogenous size distribution in the range of 20–40 nm ± 5 nm. Figure 1f shows the magnified image of the MX−Ni composite with a view of interface formation between Ti_3_C_2_T*_x_*, Ni NPs, and MXene-derived TiO_2_ (surface-oxidation). Interestingly, the Lamellar-like morphology of Ti_3_C_2_T*_x_* has undergone a substantial transformation into the crumpled structures, which might be explained based on MXene-reducing ability and strong fusion driven forces between Ni NPs [24]. Here, Ti_3_C_2_T*_x_* may act as a protective layer to Ni NPs, creating a reducing environment that results in the partial surface oxidation of Ti_3_C_2_T*_x_* to TiO_2_ (proven later) and simultaneously crumpling its layer structure owing to the electrostatic attraction between embedded Ni NPs.

**Figure 2:**
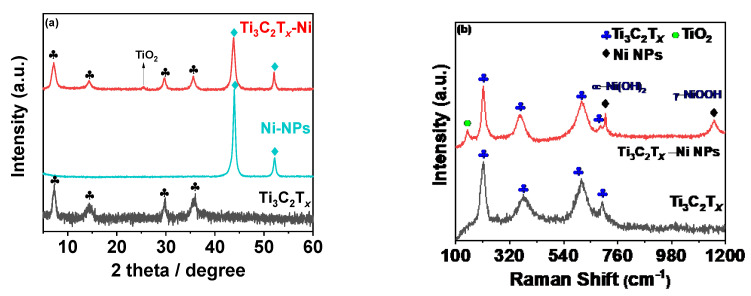
(**a**) XRD pattern recorded for MX−Ni composites in reference to pristine Ti_3_C_2_T*_x_*, and Ni NPs; (**b**) corresponding Raman spectrum with typical Ti_3_C_2_T*_x_*, Ni NPs bands with evidence of Ti_3_C_2_T*_x_* derived TiO_2_ particles.

The compositional characteristics of the synthesized composites were then assessed using XRD and Raman analysis. Figure 2a shows the XRD pattern for MX-Ni in reference to pristine Ni and Ti_3_C_2_T*_x_* counterparts. The MXene pattern consists of a typical 002 peak near 7.0° with additional peaks indexed to (004), (0010) and (0012) planes of Ti_3_C_2_T*_x_* [16]. The XRD pattern for Ni NPs consisted of two major peaks attributed to the (111) and (200) planes of FCC Ni metal as referenced against ICCD No.87-0712 [25]. No additional peaks related to oxides could be detected, confirming the purity of Ni metal as zero-valent metal nanoparticles. The MX−Ni composite exhibited characteristic peaks typical to MXene and Ni NPs with a small additional peak near 25°, indexed to the (101) plane of TiO_2_ (anatase). In addition, the typical (111) peak of Ni NPs was found to slightly shift to a lower degree, confirming the successful surface-bound interactions between Ni NPs and Ti_3_C_2_T*_x_* substrate. The occurrence of TiO_2_ is related to the modest surface oxidation of Ti_3_C_2_T*_x_* subsequent to its reductive behavior towards Ni NPs [24]. This further anticipates MXenes’ ability to function as a reducing/capping agent to metal NPs, blocking the metals (Ni) oxidation process. Since the formed TiO_2_ is relatively small, we anticipate it would have a trivial influence on the electrochemical characteristics of MXene and Ni NPs. Nonetheless, it may contribute to creating active sites without impairing the composites’ overall conductivity.

**Figure 3:**
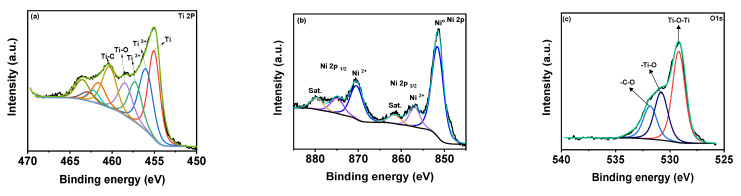
XPS high-resolution peak profiling of (**a**–**c**) Ti 2þ, Ni 2þ and O1s of MX−Ni composites.

The Raman spectra of the MX-Ni is shown in Figure 2b. The typical Raman bands of Ti_3_C_2_T*_x_* were identified at 198, 393, 630 and 710 cm^−1^ [26]. In the case of MX−Ni, typical bands of Ti_3_C_2_T*_x_* were identified along with an additional band near 144 cm^−1^ for TiO_2,_ confirming virtually little surface oxidation of MXenes. Moreover, second-order transitions corresponding to Ni(OH)_2_ were observed near 770 cm^−1^, and a band near 1150 cm^−1^ for –NiOOH confirmed the presence of Ni NPs with MXene in a composite configuration [27]. XPS analysis was used to determine the surface chemical compositions for MX−Ni composites. Figure 3a shows the XPS deconvoluted Ti2þ band energy profile of Ti_3_C_2_T*_x_* in MX−Ni composites. The typical Ti, Ti 2þ, Ti 3þ and Ti-C bond energies were identified near 455.0, 461.7, 455.9, 462.0, 457.0, 462.5 and 460 eV, respectively [28]. The bond energy near 458 eV further confirmed the formation of TiO_2_ from partial surface oxidation of Ti_3_C_2_T*_x_*. The Ni 2þ profile is shown in Figure 3b, with major bond energies corresponding to the zero-valent state of Ni (850.6 eV) and typical Ni^2+^ oxidation states. The bond energy of Ni° is relatively higher than Ni^2+^, indicating stable pure metallic Ni particles. The O1s spectra (Figure 3c) could be deconvoluted into Ti-O-Ti, and -Ti-O and –C-O bond energies near 530 eV, confirming the partial oxidation of MXene. Though traces of TiO_2_ were evident in XPS for MX-Ni composites, major phases of the composite were identified as Ti_3_C_2_T*_x_* and Ni. Thus, synergism of high current conductivity and superior redox activity could be anticipated during the electrochemical performance.

**Figure 4:**
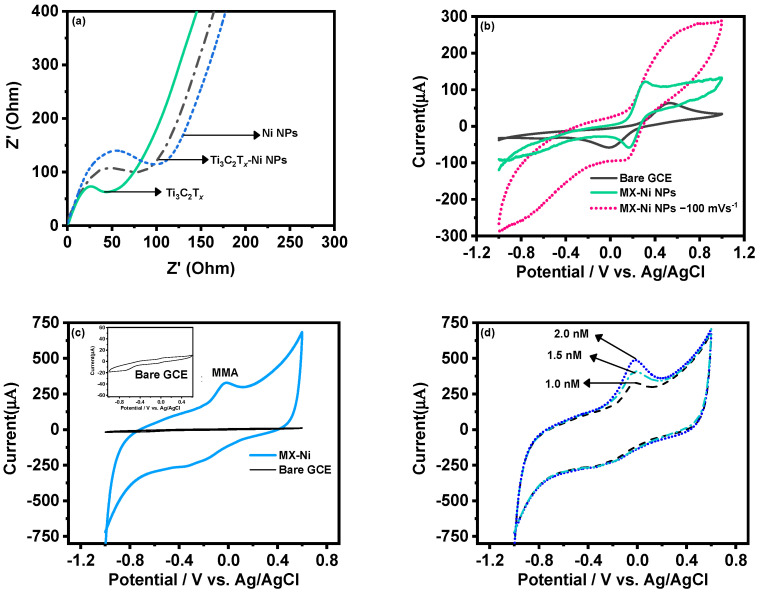
(**a**) EIS-based Nyquist plots for MX−Ni composites; (**b**) CV curves for MX−Ni composite in K_4_[Fe (CN)_6_]/K_3_[Fe (CN)_6_] in 0.1 M KCl in reference to bare-GCE and pristine Ni NPs modified GCE at 50 mVs^−1^ and 100 mVs^−1^; (**c**) the electrochemical response of MX−Ni against 0.001 µM MMA; (**d**) corresponding CV based reductive current increment observed against different concentration in the range from 1.0 to 2.0 nM MMA at the scan rate of 50 mVs^−1^.

The MX−Ni electrode was later characterized for its improved electrochemical kinetics using the EIS technique. Figure 4a shows the corresponding Nyquist plot of MX−Ni in reference to pristine Ti_3_C_2_T*_x_* and Ni NPs. Here, Ti_3_C_2_T*_x_* exhibited the smallest charge-transfer resistance (smallest semi-circle) compared to MX−Ni NPs, demonstrating a lower resistance than pristine Ni NPs. This confirmed that MX−Ni composites had adopted the conductive characteristics of both MXene and Ni NPs, and faster interfacial-charge transfer could be anticipated during the redox process. Figure 4b shows the response of the MX-Ni modified electrode against 0.5 mM of K_4_[Fe (CN)_6_]/K_3_[Fe (CN)_6_] in 0.1 M KCl as a supporting electrolyte at a scan rate of 50 mVs^−1^. Unlike the bare GCE, which had a typical redox response, the MX−Ni exhibited a superior current response which was stable even at a higher scan rate of 100 mV s^−1^. The variation in redox response of MX-Ni against different ionic strength was also evaluated by varying the concentration of neutral electrolyte KCl from 0.1 to 0.25 M. Figure S2a shows MX−Ni exhibits no appreciable voltammetric current fluctuation, demonstrating materials’ exceptional ability to preserve redox reactivity in a rich ionic solution. 

The CV curves for pristine Ni NPs and Ti_3_C_2_T*_x_* modified GCE in 0.1 M PBS at a scan rate of 50 mVs^−1^ are shown in Figure S2b. Unlike Ni NPs, which exhibit a typical redox couple for Ni (II) and Ni (III) ions, the Ti_3_C_2_T*_x_* could only display a large conductive current owing to its relatively weak redox activity in a neutral PBS system. Figure 4c shows the CV curves for MX−Ni composites against 0.001 µM of MMA in 0.1 M PBS (pH 7.0) in reference to bare GCE a fixed scan rate of 50 mVs^−1^. The bare GCE had no activity against MAA (inset Figure 4c), whereas the MX−Ni successfully produced a cathodic reduction current response near 0.0 V, which was attributed to the reduction of MMA to 2-methylpropane-1,3-diol [29]. The response was recorded for different MMA concentrations (0.001 to 0.002 µM) at 50 mVs^−1^ (Figure 3d), realizing a stable peak for each concentration confirming MX−Ni composite’s steady electrocatalytic activity towards MAA. Figure S3a shows the variation of peak current against different scan rates in the range from 50 to 110 Vs^−1^, whereas the plot of peak current against the square root of scan rate (Figure S3b) further indicated the surface redox process to be diffusion controlled. Figure S3c shows the peak current variation against different pH in the range of 3–9 for MX−Ni against MMA (0.001 µM). The pH 7 realized the maximum current response, whereas the acidic medium produced the minimal response, suggesting the optimum suitability of neutral pH for selective detection of MMA molecules.

**Figure 5:**
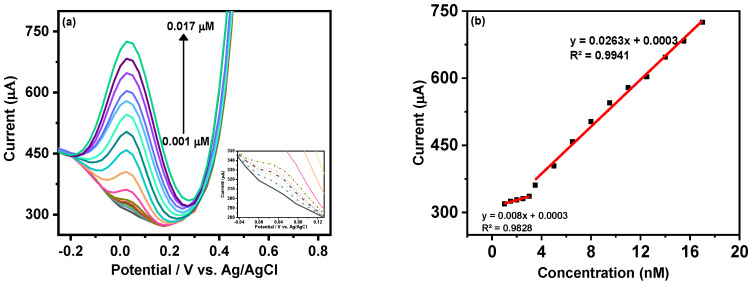

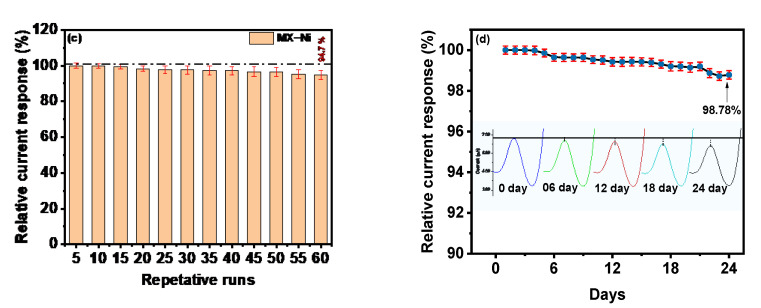
(**a**) DPV response of MX−Ni based electrode against gradually increasing concentration of MMA in the range from 0.001 to 0.017 µM with inset showing the DPV curves at lower concentration; (**b**) corresponding linear calibration curves and their linear fit analysis; (**c**) bar-graph representing the variation in DPV current response of MX−Ni electrode with an efficiency drop to 94.7% during consecutive 60 cycles; (**d**) the relative current response of single MX−Ni electrode against 0.017 µM MMA measured at the interval of 1 day for consecutive 28 days of long-term storage in deaerated sealed conditions at 4 °C with a response decline of 98.78% to its initial response along with representative DPV curves recorded at the 6th-day interval.

The analytical quantification of MMA was carried out using DPV as a primary sensing technique based on its higher sensitivity. Figure 5a shows the gradual increment in the cathodic peak current of MX−Ni composite with the rise in MMA concentration from 0.001 to 0.017 µM. The inset of Figure 5a shows that adequate DPV current could be attained even at a nanomolar concentration of MAA, which could be ascribed as the synergic outcome of the integration of highly conductive MXene with redox active Ni NPs with MX−Ni. Figure 5b shows the associated linear calibration plot where working linearities were defined for both low (0.001 to 0.003 µM) and high concentration (0.0035 to 0.017 µM) ranges. The overall detection limit (DL) for MAA using MX−Ni composite was estimated using the formula 3.3σ/S where “σ“ represents the standard deviation of a signal obtained at the lowest MAA concentration and “S” is the slope of the corresponding calibration curve. In this case, the MX−Ni composites-based sensor was sensitive down to 0.12 pM of MMA. In this case, the superior response of MX-Ni is directly attributed to the synergy of Ni NPs and Ti_3_C_2_T*_x_*-MXene, which improves the conductivity of the electrode and promotes surface redox reaction and interfacial charge transfer, enabling the generation of readable signal even at low MMA concentrations. This is particularly useful in the case of clinical biomarkers, which are difficult to quantify at low concentrations using traditional methods, such as ELISA.

The MX−Ni based electrochemical sensors offer a superior signal response in a wider dynamic range with sensitivity (limit of detection) lower than the competitive electrochemical sensors and conventional techniques (Table 1). To assess the MX−Ni-based electrode’s reliability, multiple DPV runs were recorded for a single MX−Ni electrode in a fixed concentration of MMA (0.001 µM) in 0.1 M PBS (pH 7.0). The bar graph in Figure 5c represents the fluctuation in the relative current response during 60 consecutive cycles. At a fixed potential of 0.0 V, MX−Ni exhibited a steady response with a sustained relative current response of 94.7% on the 60th cycle with an acceptable standard deviation of 1.68 to 2.0%, verifying the sensor’s superior signal repeatability.

The selectivity of a sensor is critical in evaluating promising workability in the complex biological matrix. The MX-Ni electrode was assessed for its selectivity towards MMA in the presence of various chemical species such as ascorbic acid (20 µM), glucose (7 mM), dopamine (0.065 nM), malic acid (2 µM), urea (50 mM), uric acid (0.4 mM), albumin (10 µM), glutathione (1 mM) and cations such as Na^+^ (130 mM) and K^+^ ions (5 mM). In the absence of MMA, the MX−Ni electrode exhibits no apparent response to the average concentrations of these interferents (Figure S3d). However, in the presence of MMA (1.0 nM), a readable DPV signal was obtained, indicating MX−Ni composite capability to selectivity recognize MMA (Figure S3e). Figure S3f shows a bar graph depicting the variance in relative DPV current responses for MMA (1.0 nM) in the presence of average concentrations of these interferents. Despite the high concentration of interferents, the current response recorded for MMA using MX−Ni, affords minimal fluctuation, indicating the sensor’s strong selectivity for MMA. The selectivity, in this case, is attributed to the narrow oxidation potential window of MMA (−0.1 to 0.2 V) over the MX−Ni electrode, which is inadequate to oxidize other common interferents. Moreover, the low-over potential of MX−Ni is the synergic outcome of redox activity, surface area and boosted electrochemical conductivity of Ni and Ti_3_C_2_T*_x_* self-assembled into a crumpled nanoarchitecture.

Long-term storage stability and workability are other critical parameters for sensors practical assessment. The newly fabricated MX−Ni electrode was stored in a deaerated environment under sealed conditions at 4 °C for 24 days. The long-term stability of the electrode was evaluated following its DPV current responses against MMA (0.017 µM) at an interval of 1 day during the 24 days of storage. The response efficiency of MX−Ni decreased to 99.42% of the initial response on the 12th day and about 98.78% on the 24th day (Figure 5d). The corresponding DPV curves measured at the interval of 6th-day is also shown in Figure 5, proving the MX−Ni capability to provide a consistent current response during long-term storage. The excellent response efficiency of the MX−Ni sensor confirms its potential for practical applications while paving a new route to using MXenes as a suitable interactive substrate to construct functional sensors for clinical biomarkers.

The clinical applicability of devised sensor was assessed by utilizing the MX−Ni electrode for quantification of MMA from complex biological matrices such as human urine samples. Table 2 provides the analytical reliability data obtained from the standard addition-based quantification of MMA using MX−Ni composites. The human urine samples were collected from healthy voluntaries against their informed consent. The samples were filtered and diluted to specific volumes using PBS (0.1 M), followed by spiking with a specific concentration of MMA and subjecting samples for DPV measurement. The recovery values were determined between 99 and 100.9%, confirming the devised sensor’s analytical reliability to work in a harsh biological matrix-like human urine with minimal relative standard deviation (RSD).

In general, the use of 2D-MXenes for direct self-assembly driven composites with redox active metals could become a promising avenue for exploring various electrochemical redox reactions owing to MXenes chemical versatility, adaptive surface functionality and hydrophilicity. These hybrid composites could be anticipated for superior performance in a myriad of applications, including electrocatalysis, photocatalysis and more importantly, electro (photo) chemical biosensors.

## 4. Conclusions

In summary, Ni NPs-embedded MXene-Ti_3_C_2_T*_x_* composite was used as an electrocatalytic active platform to build a simple, rapid and highly sensitive MMA sensor. Few-layer thick Ti_3_C_2_T*_x_* sheets were prepared using the conventional HF-etching approach followed by their loading with zero-valent Ni NPs, resulting in self-assembled cluster-like architectures. The self-assembly approach enabled the construction of a unique interfacial arrangement where Ti_3_C_2_T*_x_* sheets served as a conductive substrate and as a protective layer to nickel nanoparticles (Ni NPs), thereby preventing their surface oxidation and aggregation. The hybrid composites (MX−Ni), when configured into an electrochemical sensor, produced a highly sensitive cathodic reduction response against methylmalonic acid (MMA), an important biomarker to vitamin B12. The robust redox capability of Ni NPs in synergism with high conductivity of MXene gave rise to high signal sensitivity in a dynamic detection ranges including low (0.001 to 0.003 µM) and high concentration (0.0035 to 0.017 µM) of MMA, with a detection sensitivity reaching 0.12 pM of MMA. Additionally, the sensor exhibited a reproducible signal signature with reliable working capability for MMA in a complex biological matrix such as human urine samples, thereby indicating its potential for practical clinical use.

Ultimately, the proposed use of the MX-Ni composite system paves the way for an improved electrochemical sensor designed for clinical applications via employing Ti_3_C_2_T*_x_* as a possible interactive electro-active conductive substrate.

## Figures and Tables

**Table 1:** Comparison of MX-Ni-based electrochemical sensor’s analytical parameters for MMA with recently reported competitive sensors.

Technique	Active Material	Linear Range	Detection Limit	Ref.
**DPV**	Ag-PEDOT/PGE (molecularly imprinted)	0.50 pM–55 nM	0.16 pM	[29]
**DPV**	GO/AuNP-co-ATMS-g-AEMA/AA (molecularly imprinted)	0.5–3 mg/dL	0.2095 µM/L	[30]
**CV/DPV**	PdAu-PPy tailored carbon fiber paper (molecularly imprinted)	4.01 pM–52.5 nM	1.23 pM	[31]
**DPV**	**MX-Ni NPs composites**	**0.001–0.017 µM**	**0.12 pM**	**This work**

**Table 2:** Analytical reliability data for MMA detection from human urine samples using an MX-Ni composite-based electrochemical sensor.

Samples	Added (µg·mL^−1^)	Expected (µg·mL^−1^)	Detected * (µg·mL^−1^)	Recovery %	RSD %
Urine-Sample-1	1.2	6.64	6.55	0.98	0.85
Urine-Sample-2	2.0	7.45	7.52	100.9	1.02
Urine-Sample-3	2.5	8.50	8.45	99	1.12

* Mean values of three consecutive readings.

## Data Availability

No data availability.

## Supplementary Materials

The following supporting information can be downloaded at: https://www.mdpi.com/article/10.3390/bios12040231/s1, Figure S1: TEM images of (**a**) few-layer thick MXenes; (**b**) pristine Ni NPs; Figure S2: (**a**) CV curve for MX−Ni composite recorded in 0.5 mM of K_4_[Fe (CN)_6_]/K_3_[Fe (CN)_6_] with variable in ionic strength of neutral supporting electrolyte KCl from 0.1 to 0.25 M; (**b**) CV profiles of pristine Ni NPs and Ti_3_C_2_T*_x_* modified GCE in 0.1 M PBS (pH 7.0) at scan rate of 50 mVs^−1^; Figure S3. (**a**) CV profile of MX−Ni composites at different scan rates from 50 to 110 mVs^−1^ against a fixed concentration of MMA; (**b**) corresponding plot for current intensity against the square of scan rate confirming the surface-redox process to be diffusion controlled; (**c**) the variation of MX−Ni response against different pH from 3 to 9 showing optimum MMA response at biological pH of 7; (**d**) the DPV response of MX−Ni electrode for different interferents without MMA; (**e**) the corresponding DPV response against 0.001 µM of MMA; (**f**) the variation in DPV current response of MX−Ni electrode for MMA (0.001 µM) in the presence of interferents such as ascorbic acid (20 µM), glucose (7 mM), dopamine (0.065 nM), malic acid(2 µM), urea (50 mM), uric acid (0.4 mM), albumin(10 µM), glutathione(1 mM) and cations such as Na^+^ (130 mM) and K^+^ ions (5 mM).
