# Supplementary material for: Ni Nanoparticles Embedded Ti3C2Tx-MXene Nanoarchitectures for Electrochemical Sensing of Methylmalonic Acid
Source: Biosensors (Basel). 2022 Apr 10;12(4):231. doi: 10.3390/bios12040231 (PMC9030921; doi:10.3390/bios12040231)
Supplement: Supplementary file 1 [file biosensors-12-00231-s001.zip › biosensors-1651633-supplementary.pdf]

biosensors

Supplementary Data

Ni Nanoparticles Embedded $Ti_3C_2T_x$-MXene Nanoarchitectures for Electrochemical Sensing of Methylmalonic Acid

Jai Kumar [1], Razium Ali Soomro [1,*], Rana R. Neiber [2,3], Nazeer Ahmed [4], Shymaa S. Medany [5], Munirah D. Albaqami [6] and Ayman Nafady [6]

1 State Key Laboratory of Organic-Inorganic Composites Beijing Key Laboratory of Electrochemical Process and Technology for Materials, Beijing University of Chemical Technology, Beijing 100029, China; 2020420024@mail.buct.edu.cn
2 Beijing Key Laboratory of Ionic Liquids Clean Process, CAS Key Laboratory of Green Process, and Engineering, Institute of Process Engineering, Chinese Academy of Sciences, Beijing 100190, China; rana@ipe.ac.cn
3 College of Chemical Engineering, University of Chinese Academy of Sciences, 19 A Yuquan Road, Beijing 100049, China
4 Research Center on Nanotechnology Applied to Engineering of Sapienza (CNIS), Sapienza University of Rome, P. le Aldo Moro 5, 00185, Rome, Italy; ahmed.1875130@studenti.uniroma1.it
5 Department of Chemistry, Faculty of Science, Cairo University, Cairo, Giza 12613, Egypt; shymaaamir80@cu.edu.eg
6 Department of Chemistry, College of Science, King Saud University, P.O. Box 2455, Riyadh 11451, Saudi Arabia; muneerad@ksu.edu.sa (M.D.A.); anafady@ksu.edu.sa (A.N.)
* Correspondence: 2021700008@mail.buct.edu.cn; Tel.: +86-178-0101-4094

Citation: Kumar, J.; Soomro, R.A.; Neiber, R.R.; Ahmed, N.; Medany, S.S.; Albaqami, M.D.; Nafady, A. Ni nanoparticles Embedded $Ti_3C_2T_x$-MXene Nanoarchitectures for Electrochemical Sensing of Methylmalonic Acid. *Biosensors* **2022**, *12*, 231. https://doi.org/10.3390/bios12040231

Received: 9 March 2022
Accepted: 8 April 2022
Published: 10 April 2022

Publisher's Note: MDPI stays neutral with regard to jurisdictional claims in published maps and institutional affiliations.

Copyright: © 2022 by the authors. Submitted for possible open access publication under the terms and conditions of the Creative Commons Attribution (CC BY) license (https://creativecommons.org/licenses/by/4.0/).

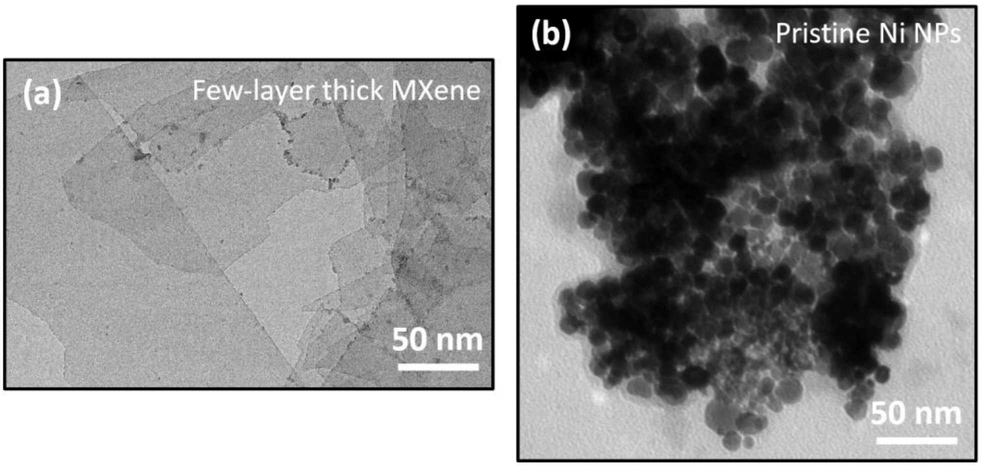

Figure S1. TEM images of (**a**) few-layer thick MXenes; (**b**) pristine Ni NPs.

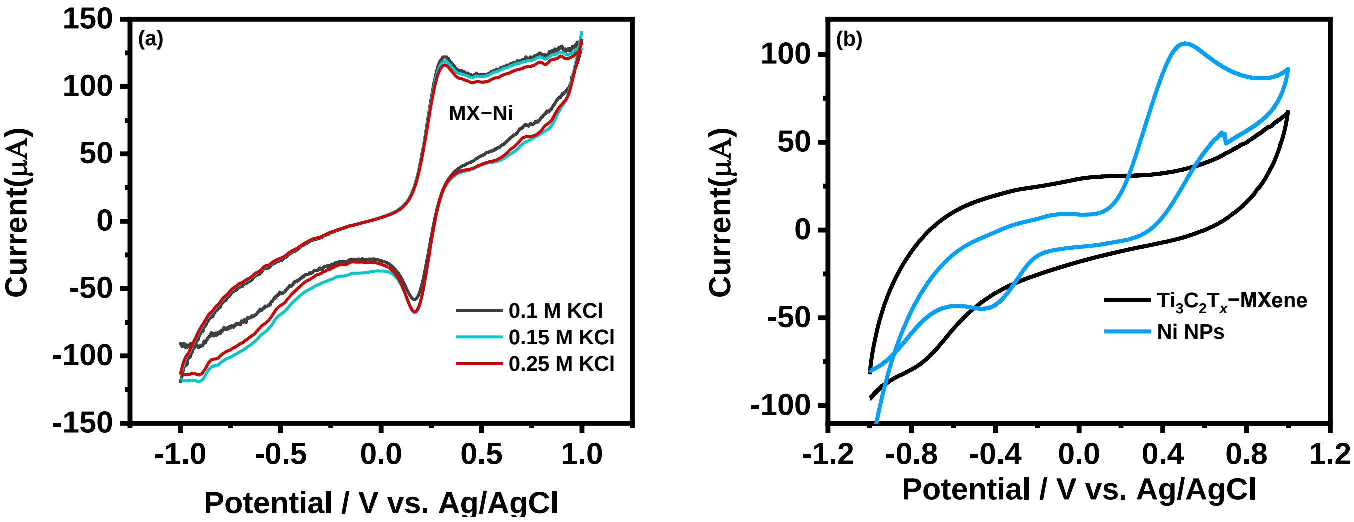

Figure S2. (**a**) CV curve for MX−Ni composite recorded in 0.5 mM of $K_4[Fe(CN)_6]/K_3[Fe(CN)_6]$ with variable in ionic strength of neutral supporting electrolyte KCl from 0.1 to 0.25 M; (**b**) CV profiles of pristine Ni NPs and $Ti_3C_2T_x$ modified GCE in 0.1 M PBS (pH 7.0) at scan rate of 50 mVs^{-1}.

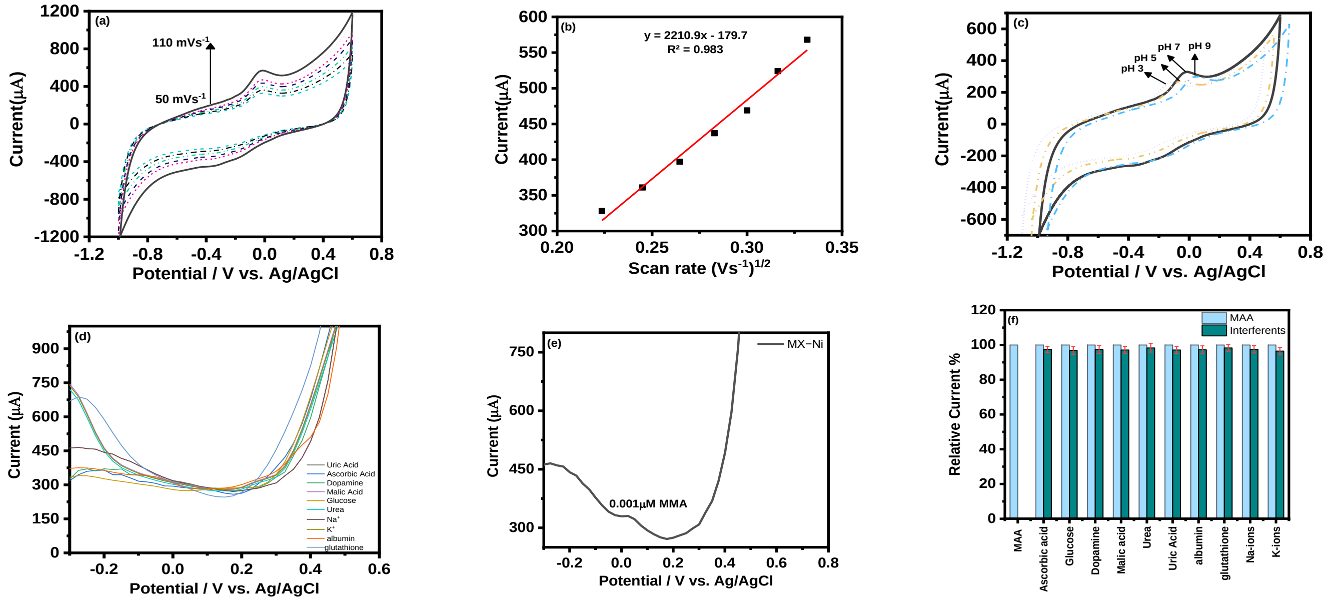

Figure S3. (**a**) CV profile of MX−Ni composites at different scan rates from 50 to 110 mVs^{-1} against a fixed concentration of MMA; (**b**) corresponding plot for current intensity against the square of scan rate confirming the surface-redox process to be diffusion controlled; (**c**) the variation of MX−Ni response against different pH from 3 to 9 showing optimum MMA response at biological pH of 7; (**d**) the DPV response of MX−Ni electrode for different interferents without MMA; (**e**) the corresponding DPV response against 0.001μM of MMA; (**f**) the variation in DPV current response of MX−Ni electrode for MMA (0.001 μM) in the presence of interferents such as ascorbic acid (20 μM), glucose (7 mM), dopamine (0.065 nM), malic acid(2 μM), urea (50 mM), uric acid (0.4 mM), albumin(10 μM), glutathione(1 mM) and cations such as Na^+ (130 mM) and K^+ ions (5 mM).
